# Investigation of the Average Shape and Principal Variations of the Human Talus Bone Using Statistic Shape Model

**DOI:** 10.3389/fbioe.2020.00656

**Published:** 2020-07-02

**Authors:** Tao Liu, Nadr M. Jomha, Samer Adeeb, Marwan El-Rich, Lindsey Westover

**Affiliations:** ^1^Department of Mechanical Engineering, University of Alberta, Edmonton, AB, Canada; ^2^Department of Mechanical Engineering, Khalifa University, Abu Dhabi, United Arab Emirates; ^3^Department of Surgery, University of Alberta, Edmonton, AB, Canada; ^4^Department of Civil and Environmental Engineering, University of Alberta, Edmonton, AB, Canada

**Keywords:** talus implant design, groupwise registration, statistical shape model, principal component analysis, geometric analysis

## Abstract

Due to the complexity of articular interconnections and tenuous blood supply to the talus, talus fractures are often associated with complications (e.g., avascular necrosis). Currently, surgically fusing the talus to adjacent bones is widely used as treatment to talus fractures, but this procedure can greatly reduce mobility in the ankle and hindfoot. Alternatively, customized talus implants have shown an overall satisfactory patient feedback but with the limitation of high expenses and time-consuming manufacturing process. In order to circumvent these disadvantages, universal talus implants have been proposed as a potential solution. In our study, we aimed to develop a methodology using Statistical Shape Model (SSM) to simulate the talus, and then evaluate the feasibility of the model to obtain the mean shape needed for universal implant design. In order to achieve this, we registered 98 tali (41 females and 57 males) and used the registered dataset to train our SSM. We used the mean shape derived from the SSM as the basis for our talus implant template, and compared our template with that of previous works. We found that our SSM mean shape talus implant was geometrically similar to implants from other works, which used a different method for the mean shape. This suggests the feasibility of SSM as a method of finding mean shape information for the development of universal implants. A second aim of our study was to investigate if one scalable talus implant can accommodate all patients. In our study, we focused on addressing this from a geometric perspective as there are multiple factors impacting this (e.g., articular surface contact characteristics, implant material properties). Our initial findings are that the first two principal components should be afforded consideration for the geometrical accuracy of talus implant design. Additional factors would need to be further evaluated for their role in informing universal talus implant design.

## Introduction

The talus plays a critical role transmitting load during weight bearing activities as it is the functional connector between the leg and the foot. It controls ambulation due to its unique and complex shape, and large articular surface (70%) (Summers and Murdoch, 2012; Taniguchi et al., 2015). Talus fractures often lead to complications such as avascular necrosis, nonunion and arthritis due to its complex articular interconnections and tenuous blood supply. These complications result in pain, stiffness, and long-term disability (Saltzman et al., 2006; Barton et al., 2011). A common treatment for sequelae of talus fractures is surgical fusion of the talus to one or more of its surrounding bones, most commonly the tibiotalar (ankle) joint or the talocalcaneal (subtalar) joint or both. This results in severe loss of motion of the ankle and hindfoot joints. Additionally, the surgical treatment is technically difficult and can lead to high rates of complications (Barg et al., 2015; Lawton et al., 2017). Furthermore, when the primary cause of joint deterioration is avascular necrosis of the talus with collapse of the talus bone, the only viable surgical solution has been a tibio-talar-calcaneal (TTC) fusion (a combined ankle and subtalar joint fusion). This can result in adequate pain relief but severe ankle and hindfoot stiffness, making ambulation difficult. Due to the significant functional limitations of this surgical procedure, some surgeons have developed custom talar bone implants using metal or ceramic for these patients (Tanaka et al., 2003; Taniguchi et al., 2015; Katsui et al., 2019).

When it comes to the design of a customized talus implant, a CT scan with 1 mm cuts of the healthy unaffected talus is performed (Islam et al., 2014). The image is segmented and an STL file is created and then mirrored, which is followed by either milling or 3D printing of the talar prosthesis. Clearly this process is possible as noted by many published reports (Harnroongroj and Vanadurongwan, 1997; Tanaka et al., 2003; Stevens et al., 2007; Taniguchi et al., 2012, 2015; Harnroongroj and Harnroongroj, 2014). However, this design process, including steps from 3D geometry preparation to external implant manufacturing can take many weeks to months based on surgical experience, which increases the time between surgical treatment decision to proceed with talar replacement and the actual surgical procedure. Traditional subtractive manufacturing techniques such as multi-axis CNC milling can be expensive and the creation of the appropriate computer files and review of the talus design (to remove bone abnormalities and irregularities) can be time-consuming. More recent innovations such as additive manufacturing including 3D printing have changed the actual processing of the implant but this does not change the workup required to have an appropriate file ready to print (Bowes et al., 2019).

Previous studies have attempted to capture the inter-individual variability of the talus by measuring a limited number of parameters. For example, this has been done via direct measurement of 55 dried human talus bones (Mahato, 2011); via measurement of two dimensional parameters through x-ray of 36 subjects (Stagni et al., 2005); or via 3D CT images of the talus (Hayes et al., 2006; Wiewiorski et al., 2016). (Trovato et al., 2017) recently quantified detailed 3D deviations among talus bones and determined that talus shape is similar between patients and that a universal talus implant in various sizes could appropriately fit the vast majority of patients. Initially, Islam et al. (2014)'s 3D deviation analysis of 27 patients showed that all tali are geometrically similar, and thus proposed to a series of five implant sizes to represent a wide range of subjects using the scale of one talus template. Subsequently, Trovato et al. (2017) used the same method, but expanded the subject size to 91 (50 males and 41 females), and created a series of 10 unisex talus implants.

Although these previous studies have provided valuable insight into the geometric design of talus implants, they all involve heavy manual work and are prone to error (van de Giessen et al., 2012), and they are unable to provide the talus shape space that contains detailed shape variations. To overcome the above issues, SSM has been proposed as a promising way to describe shapes with geometric similarities, so it is appropriate for bones like the talus (Sarkalkan et al., 2014; Ambellan et al., 2019). Melinska et al. (2017) developed SSMs of the cuboid, navicular, and talus bones using the spherical harmonics based on 30 subjects and showed the clinical potential of SSM in representing tarsal bones. Feng et al. (2018) compared the talus bones in clubfeet and in normal feet through two methods: direct measurement and SSM; both approaches revealed differences between normal feet and clubfeet. Most recently, Tümer et al. (2019) analyzed shape variations and symmetry of the fibula, tibia, talus, and calcaneus using a nonrigid groupwise registration algorithm (van de Giessen et al., 2012), and found that all types of bones showed a similar shape pattern and are directionally symmetrical for both sides based on a dataset of 66 subjects. While these studies have pointed toward the feasibility of SSM in characterizing bone shapes, there is limited knowledge on the benefits of using SSM to design universal talus implants.

Thus, this study aimed to develop a methodology using SSM and then evaluate the feasibility of the model in acquiring the mean shape needed for universal talus implant design, and investigate if one scalable talus implant template would be able to accommodate all talar surgery patients. The development of universal talus implants can not only shorten the time between injury and surgery, but would also significantly reduce the cost compared to customized talus implants The SSM obtained can be used in the design of the universal talus implants by providing information on variation directions, implant size ranges, and can ultimately be used to inform the design of the universal talus implants.

## Materials and Methods

### 3D Geometry Acquisition

CT scans of 98 (41 females and 57 males) healthy and intact talus bones were obtained from the University of Alberta Hospital with University of Alberta Ethics Board approval. The subjects were composed of 41 females (age 43.5 ± 14.7 years) and 44 males (age 32.1 ± 15.5 years). Of the 44 male subjects, 13 contributed both a left and a right talus, thereby totaling 57 male tali. None had evidence of talus fractures. A Somatom Definition Flash Scanner was used in the study with a specification of 0.8 mm pitch, 0° gantry tilt, 80 mA current and 120 kVp voltage. Each data set contained up to 119 slices with a constant thickness of 0.6 mm, a pixel size of 0.388 mm and an image size of 512 × 512 pixels. These images were processed using the software MIMICS (Materialize NV, Belgium) and cleaned from spikes using the software Geomagic Studio 2013 (Geomagic®, Morrisville, North Carolina) in order to obtain high quality 3D geometry of the talus bones. The current study used Trovato et al. (2017)'s dataset (50 males and 41 females), however this study excluded a few low-quality male tali and included both the left and the right talus of 13 subjects. All left tali were mirrored and grouped together with the right talus bones.

### Groupwise Registration

The groupwise registration process used in this study is an expansion and modification of van de Giessen et al.'s (van de Giessen et al., 2012) work. When it comes to registration, a random talus was first selected as a reference shape for the talus group. In order to reduce the registration time of the algorithm, the 3D geometries of talus bones were aligned to this reference shape first in Geomagic Studio 2013 (Geomagic®, Morrisville, North Carolina) using the built-in best-fit alignment command. All the bones were then exported in the format of binary STL files.

**Figure 1:**
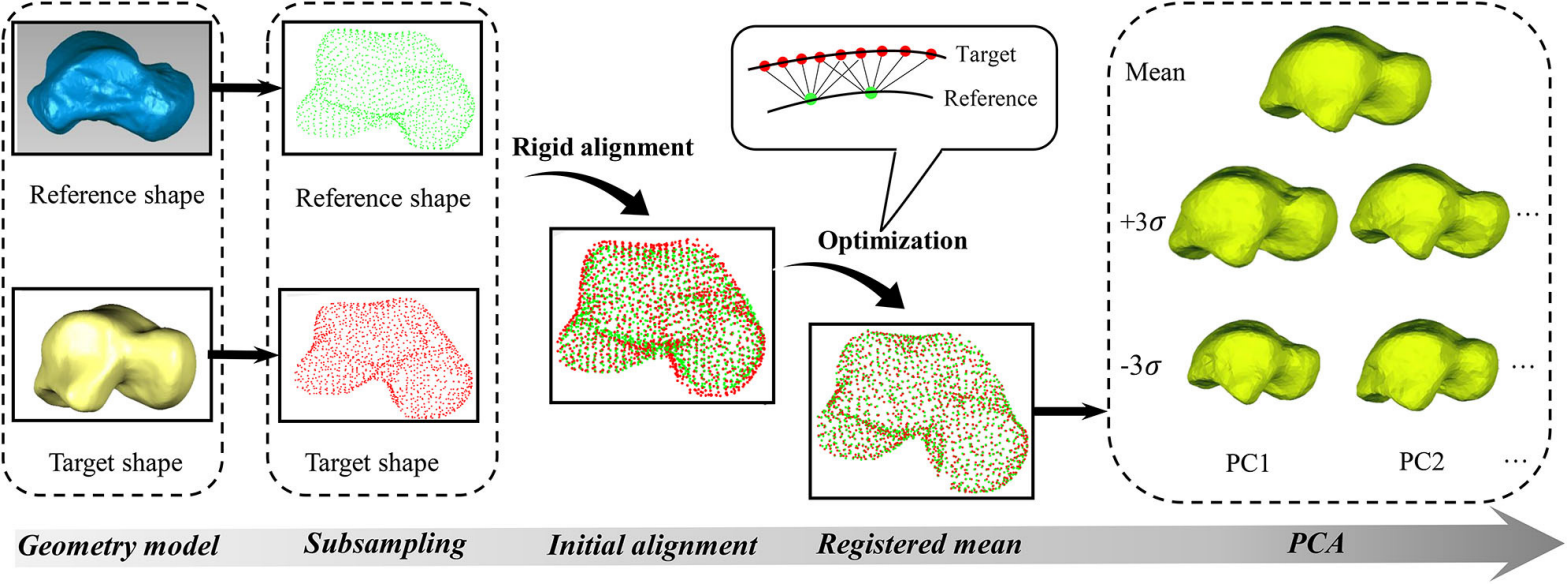
The flowchart of groupwise registration and principal component analysis (PCA).

After preparing the geometric models, both the reference and target talus shapes were represented as point clouds (***T***_***i***_). These point clouds were first subsampled in MATLAB® R2018b (MathWorks Inc., USA) using a built-in function (“pcdownsample” with “gridAverage” option) in order to reduce computational complexity (Figure 1). A rigid alignment was then performed by overlapping the geometric centers of these subsampled points, after which they were modeled using Gaussian mixture models (van de Giessen et al., 2012). In the current work, all Gaussian kernels were isotropic and had the same size, with size being determined by parameter ***σ***. For the purpose of convenience, an identity matrix of size 3 was used in current study. The density function *D*_*i*_ of each point cloud (***T***_***i***_) at coordinate *x* is expressed by Equation (1)

(1) $$\begin{array}{l} D_{i}\left(x\right)=\frac{1}{n_{i}{\left(2\pi \right)}^{3/2\;}|\sigma |\;}\overset{n_{i}}{\underset{j=1}{\sum }}\exp\left(-{\left(x-p_{ij}\right)}^{T}\sigma^{-1}\left(x-p_{ij}\right)\right) \end{array}$$

Where: *n*_*i*_ is the number of points in the talus point cloud (***T***_***i***_), ***p***_***ij***_ is the coordinates of a point indexed by *j* in the talus point cloud (***T***_***i***_), ***σ*** is the covariance matrix (In this study, ***σ*** = $\left[\begin{array}{lll} 1 & 0 & 0\\ 0 & 1 & 0\\ 0 & 0 & 1 \end{array}\right]$), ***σ***^−1^is the inverse of covariance matrix, and |***σ***| is the determinant of the covariance matrix. The *L2* divergence is generally used as a distance measure between two density functions (Wang et al., 2009). For any two clouds (***T***_***i***_ and ***T***_***j***_), the distance is defined by Equation (2)

(2) $$\begin{array}{l} f_{L2}\left(T_{i},T_{j}\right)=\int_{R^{3}}\left(D_{i}^{2}-2D_{j}D_{i}+D_{j}^{2}\right)dx \end{array}$$

Where: *D*_*i*_ is the density function for talus point clouds indexed by *i*, *D*_*j*_ is the density function for talus point clouds indexed by *j*. The *L2* divergence is symmetric and has a closed form (Equation 3) for two Gaussian density functions (Chui et al., 2004). As calculating *L2* divergence is time-consuming and only closest points have significant effects on it based on Equation (3), in order to further reduce computational complexity, the current study was limited to the six nearest neighbor points instead of the entire point clouds (Figure 1).

(3) $$\begin{array}{l} \int_{R^{3}}G\left(x|u_{1},\;\sigma_{1}\right)G\left(x|u_{2},\;\sigma_{2}\right)\;dx\;=\;G\left(0|u_{1}-u_{1},\sigma_{1}+\sigma_{2}\right) \end{array}$$

Where: *G*(***x***|***u***_**1**_, ***σ***_**1**_) and *G*(***x***|***u***_**2**_, ***σ***_**2**_) are Gaussian density functions with means ***u***_**1**_ and ***u***_**2**_, and covariance matrices ***σ***_**1**_ and ***σ***_**2**_, respectively.

In order to register the reference talus to the target talus, the transformation from the reference talus to the target talus was depicted using thin-plate-spline transform with *n*^ϕ^ control points (Bookstein, 1989). The optimization function (Equation 5) was the sum of Equations (2, 4). This function can maximize the similarity between the reference model (***T***_***i***_) and the target model (***T***_***j***_) through *L2* divergence (Equation 2), while minimizing the deformation between the reference shape and the target shape through the stress energy as produced by thin-plate-spline transform (Equation 4). In the end, the target model (***T***_***j***_) can be optimally expressed as a function of the reference shape (***T***_***i***_) by using ***T***_***j***_ = ***T***_***i***_+ ***ϕθ***_***j***_. Where: ***ϕ*** is the *n*^*i*^ × *n*^ϕ^ matrix that includes the radial basis functions of the thin-plate-spline transformation (6), and ***θ***_***j***_ is the *n*^ϕ^ × 3 matrix of coefficients associated with ***T***_***j***_.

(4) $$\begin{array}{l} f_{stress}\left(\theta_{i},T_{i}\right)=tr\left(\theta_{i}^{T}\phi^{T}\phi \theta_{i}\right) \end{array}$$

(5) $$\begin{array}{l} F\left(\theta_{1},\cdots ,\theta_{N},T_{i}\right)=\underset{{\theta_{i}\in R}^{n\phi_{\times 3}}}{\min}\sum_{i=1}^{N}\left[f_{L2}\left(T_{i},T_{i}\right)+\lambda f_{stress}\left(\theta_{i},T_{i}\right)\right] \end{array}$$

(6) $$\begin{array}{l} \phi \left(m,n\right)=\|T_{j}^{m}-T_{i}^{n}{\|}_{2} \end{array}$$

Where: λ is a scalar and taken as a regularization weight, *f*_*L*2_(***T***_***i***_, ***T***_***i***_) is a scalar function of ***T***_***i***_ and ***T***_***j***_, *f*_*stress*_(***θ***_***i***_, ***T***_***i***_) is a scalar function of ***θ***_***j***_ and ***T***_***i***_, *N* is the total number of target talus bones, m refers to the mth point in ***T***_***j***_, and *n* refers to the *n*th point in ***T***_***i***_, || ||_2_ is the Euclidean norm.

In the current work, groupwise registration was performed in MATLAB® R2018b (MathWorks Inc., USA). We solved the problem of optimization using a limited memory BFGS method (Liu and Nocedal, 1989). In order to use this method, analytic gradients were required to increase the speed of the solver convergence. Thus, the derivatives of the optimization function with respect to ***θ***_***i***_ is a sum of Equations (7, 8).

(7) $$\begin{array}{l} \frac{df_{L2}\left(T_{i},T_{j}\right)}{d\theta_{i}}=\frac{1}{n_{i}n_{j}}\overset{n_{i}}{\underset{i=1}{\sum }}\overset{n_{j}}{\underset{j=1}{\sum }}(\frac{\partial G\left(0|u_{i}^{R}-u_{j}^{R},\sigma_{i}^{R}+\sigma_{j}^{R}\right)}{\partial \theta_{i}}\\ \;-2\frac{\partial G\left(0|u_{i}^{R}-u_{j}^{T},\sigma_{i}^{R}+\sigma_{j}^{T}\right)}{\partial \theta_{i}}) \end{array}$$

Where: *n*_*i*_ is the number of points in the reference point clouds (***T***_***i***_), *n*_*j*_ is the number of points in the target point cloud (***T***_***j***_), $u_{i}^{R}$ is the mean indexed by *i* in the reference point cloud, $\sigma_{i}^{R}$ is the covariance matrix indexed by *i* in the reference point cloud, $u_{j}^{T}$ is the mean indexed by *j* in the reference point cloud, $\sigma_{j}^{T}$ is the covariance matrix indexed by *j* in the reference point cloud.

(8) $$\begin{array}{l} \frac{df_{stress}\left(\theta_{i},T_{i}\right)}{d\theta_{i}}=2\phi^{T}\phi \theta_{i} \end{array}$$

After the reference shape was registered using Equation 5, the average distance between the points in the registered shape and their corresponding closest points in the target shape was used to measure if the registered talus bone was within a predefined tolerance level. When it was not, the results were further optimized using Equation 5 by separately minimizing the points that have a relatively larger deviation from the target shape in the previous iteration. This process was repeated until the overall distance fell within the predefined tolerance. Once the groupwise registration for all tali was done, principal component analysis (PCA) was then performed based on the registered talus bones to obtain the average shape and the associated principle shape variations in MATLAB. This study performed PCA for the males and females separately. For the convenience of visualization and comparison, all the point clouds were triangulated in MATLAB.

## Results

### Algorithm Accuracy

**Figure 2:**
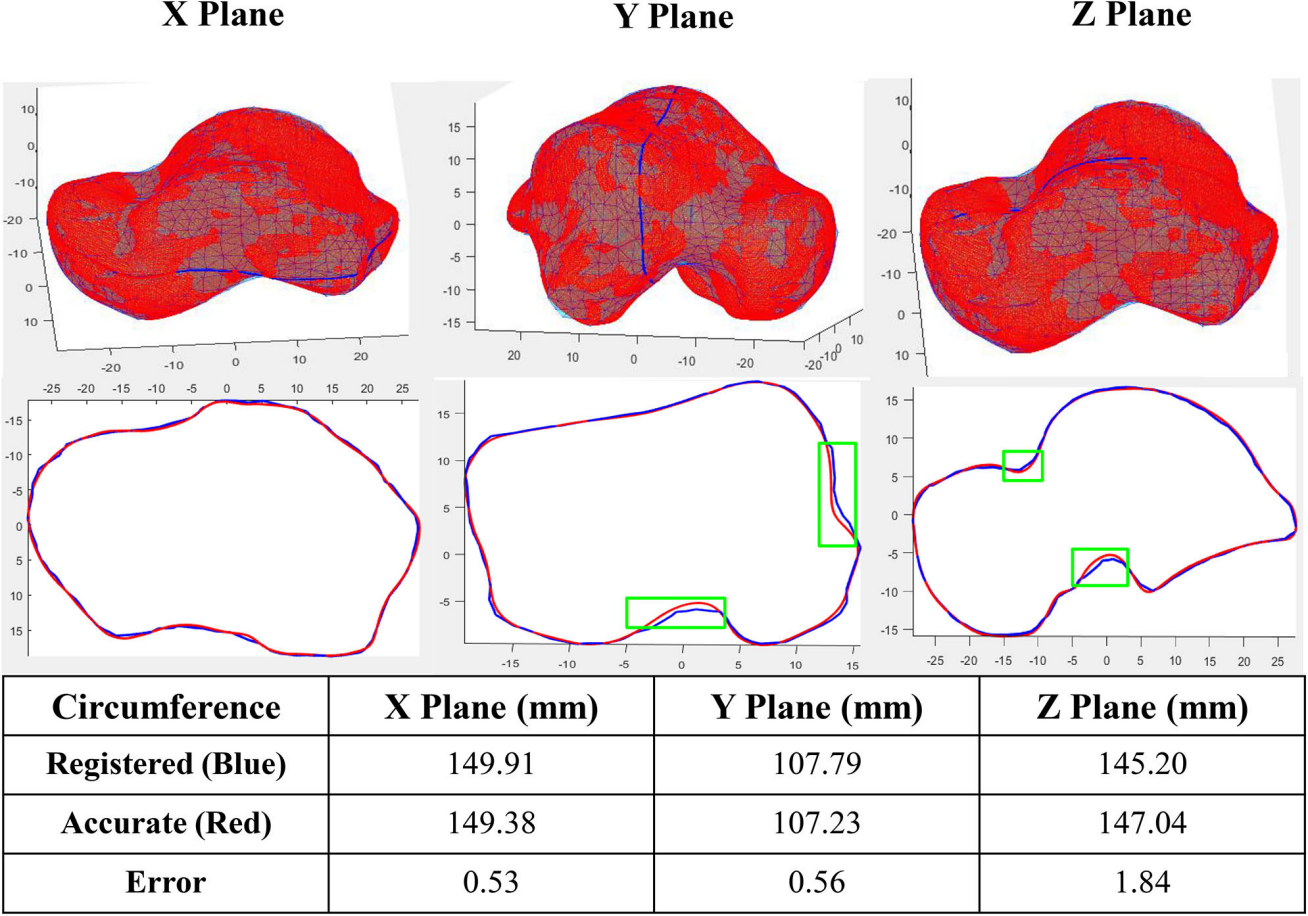
Accuracy of a sample registration (Green box shows the location of maximum circumference variations in three planes).

Sample tests were performed first in order to quantify the accuracy of the algorithm. A registered talus bone was randomly selected and compared to its target shape in three perpendicular planes. The curves in each plane were obtained by intersecting the two tali (the registered and target) with the X, Y, and Z planes respectively (Figure 2). With the algorithm setting of 1,360 points in reference shape, around 2,000 points in each target shapes, 0.4 mm for predefined tolerance and 10^−11^ for regularization weight (lambda), the maximum circumference difference was 1.84 mm, which occurred in the Z plane. Both the X plane and Y plane had a similar error value of ~0.5 mm. In addition, the maximum circumference variations were located at the non-articular surfaces.

### Principal Component Analysis (PCA)

**Figure 3:**
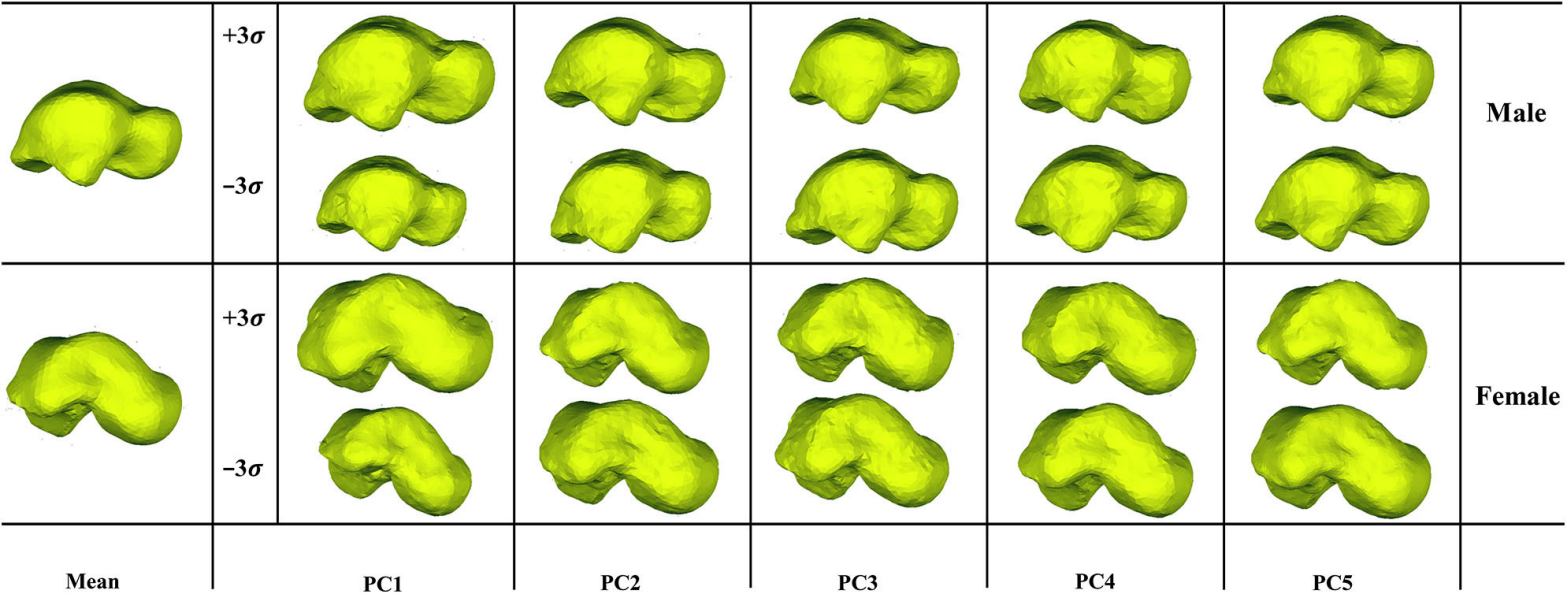
Visualization of the first five PCA eigenvectors scaled by ±3σ for male and female tali.

We examined the first five PCA components for both male and female tali. Our findings showed that the first major shape variation was related to the tali size of both females and males, while the second largest shape variation was highly associated with articular surfaces of the talus. For other principal components, shape variations lay in local surfaces (Figure 3).

### Deviation of Principal Shape Variations

**Figure 4:**
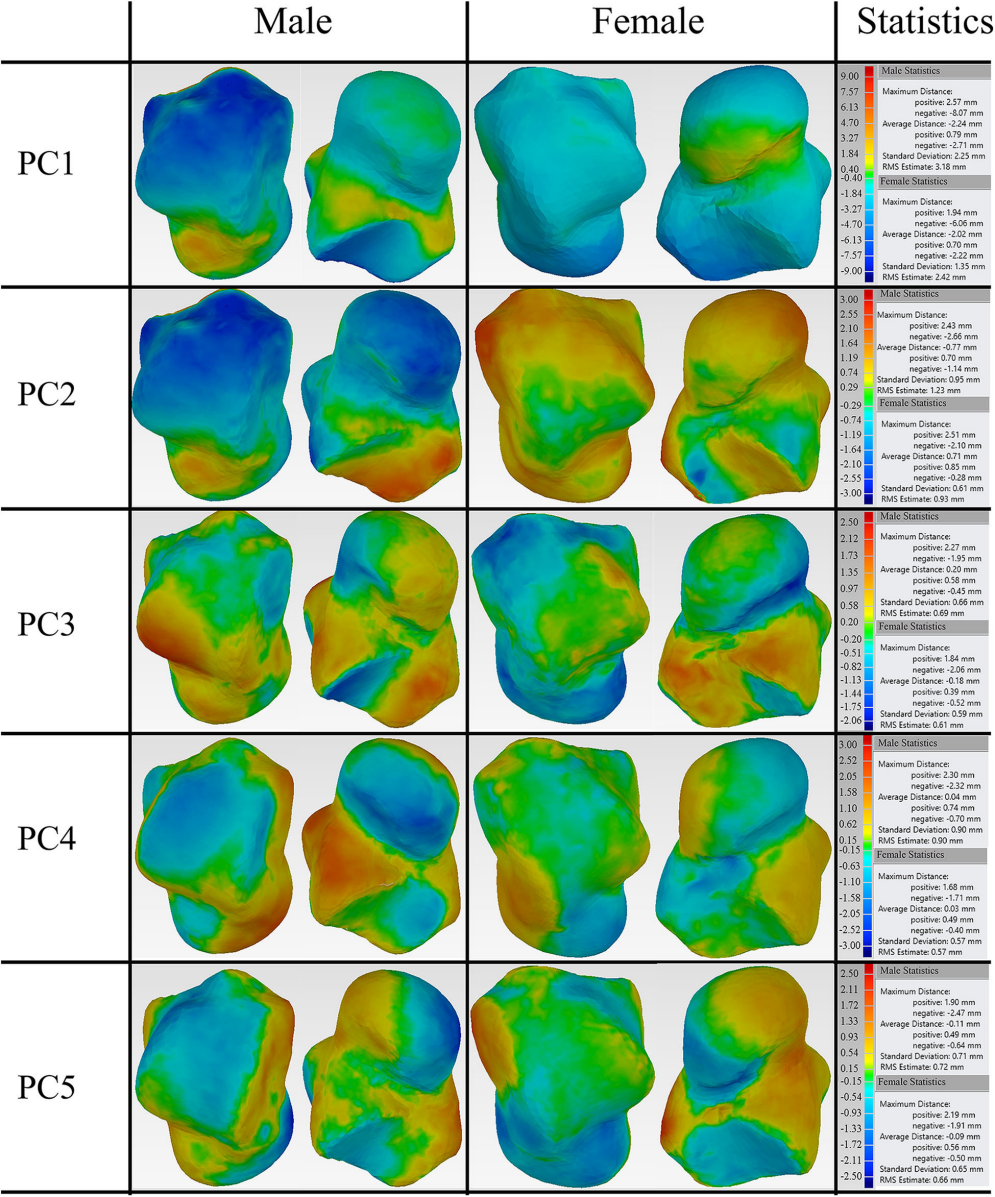
Deviation analysis between the first five PCA eigenvectors scaled by +3σ and their mean shape for both male and female tali.

The first five principal talus shape variations (+3σ) were also quantitatively compared with their mean shapes (Figure 4). For both males and females, PC1 had the largest average distance with a magnitude of ~2.1 mm; PC2 had the second largest average distance and a magnitude of ~0.7 mm. This is followed by PC3, which had a value of ~0.2 mm. The rest of the principal variations showed a relatively smaller magnitude (≤0.1 mm).

**Figure 5:**
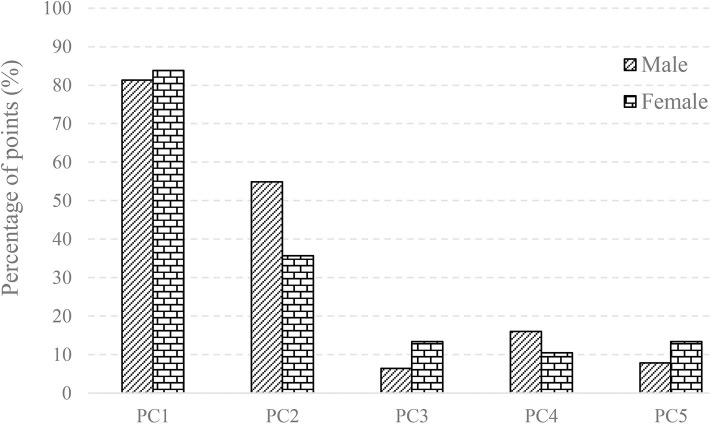
The percentage of points with the deviation >1 mm in the first five PCA eigenvectors scaled by +3σ and its mean shape for male and female.

Furthermore, the percentage of points that deviate more than 1 mm was quantified for the first five major shape variations with respect to their mean shapes. Our findings revealed that both the average male and female talus followed a similar trend in the first five major shape variations. Specifically, PC1 had the highest percentage of points (>80%), and PC2 had the second highest percentage (55% for males and 36% for females). This is in contrast to the three other major shape variations, whose percentage ranged from 6.4 to 16% (Figure 5).

### Geometry Comparison With Other Studies

In the current study, male and female tali were registered separately, and used to train two separate SSMs, thereby producing two sex-specific mean talus shapes in the end. These two mean shapes were used for the design of two sex-specific talus implant templates and compared with other talus implants in the literature (Trovato et al., 2017; Bowes et al., 2019). Using the male talus implant shape as a reference, we compared three talus shapes. The first comparison is with the talus implant designed by Trovato et al. (2017)'s study, who based their results on 91 subjects. The second one is a universal talar implant (Bowes et al., 2019) that has been modified by an experienced surgeon with 20 years of clinical experience based on Trovato et al. (2017)'s original design, from here on referred to as “surgeon-altered” talus for convenience. The last one is the average female talus implant from the current study. All geometries were scaled to the same volume (33,540 mm^2^) that Trovato et al. (2017)'s average shape has before comparison.

**Figure 6:**
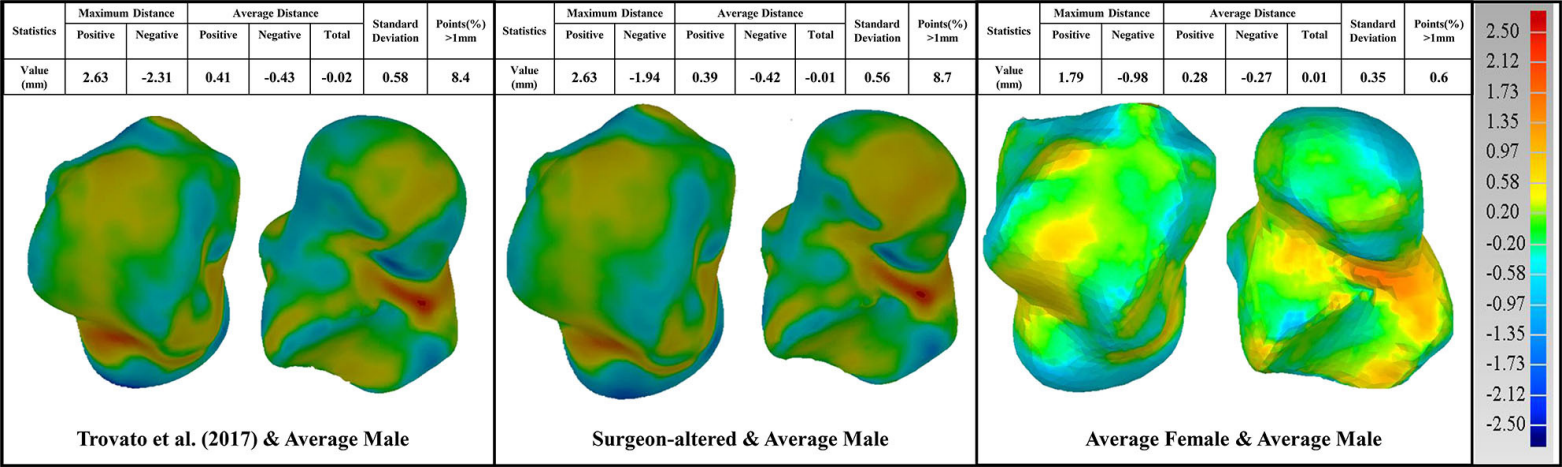
Comparison of the average male talus in the study (as reference), with average talus shapes from three sources: (1) Trovato et al. (2017); (2) surgeon-altered; (3) the female talus in the current study.

3D deviation analysis was performed using Geomagic comparing the reference to the aforementioned three talus shapes. First, when comparing the reference male talus with the surgeon-altered one, we found relatively larger maximum positive and negative deviations of 2.63 and −1.94 mm respectively. Second, when the reference was compared to Trovato et al. (2017)'s average shape, a similar maximum deviation was found at 2.63 mm for the positive and −2.31 mm for the negative. Lastly, comparing the reference and the female talus average from the present study, in contrast with the first two comparisons, we found a lower maximum deviation at 1.79 and −0.98 mm. Overall, the value of average distance for all comparisons was ~0.015 mm (Figure 6).

In these three comparisons, the percentage of points with deviations >1 mm were also quantified. When compared to the male talus shape, surgeon-altered and Trovato et al. (2017)'s talus showed a similar percentage at 8%, whereas the average female shape had the lowest percentage at 0.6% (Figure 6).

**Figure 7:**
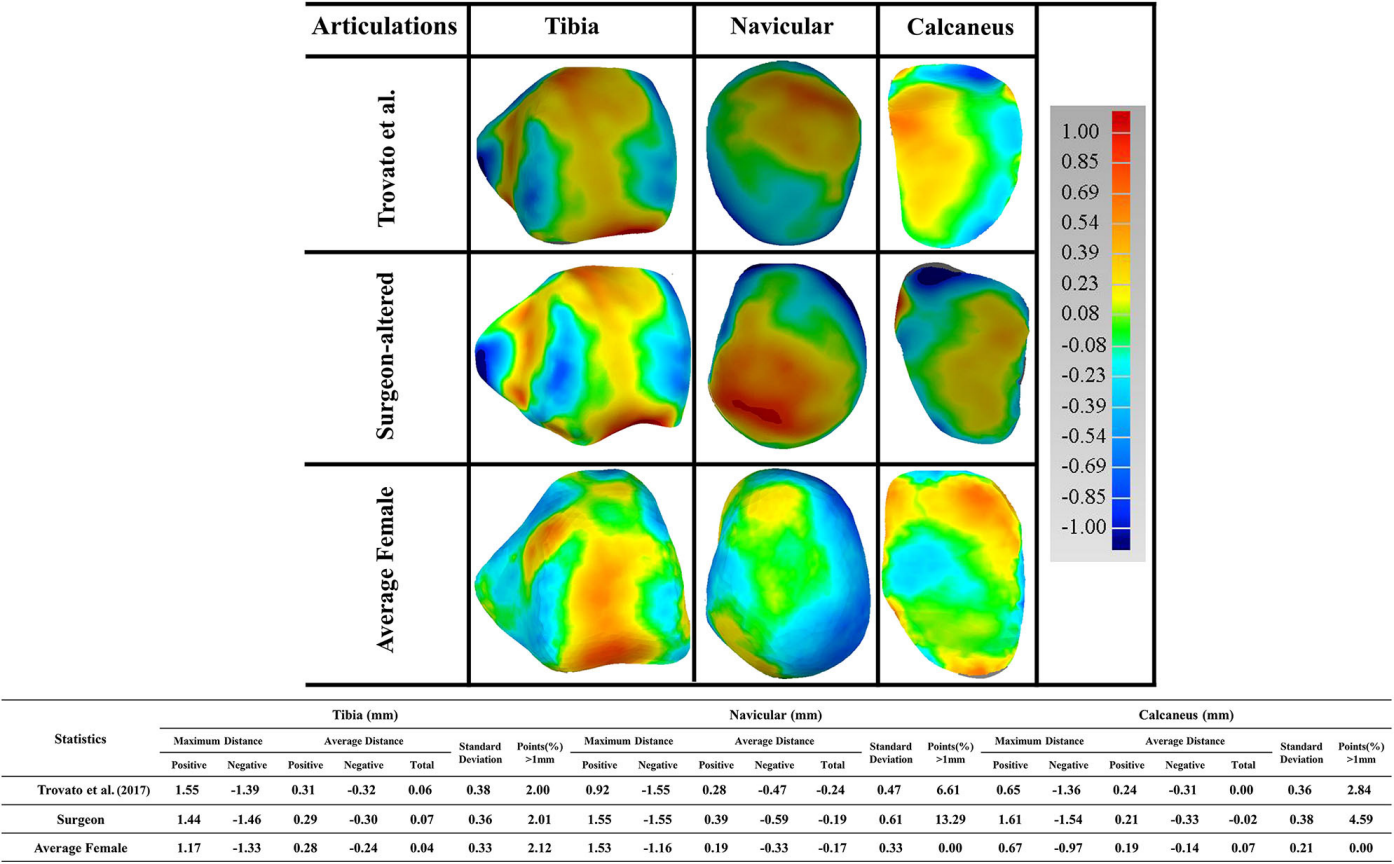
Comparison of the male talus articulations in the study (as reference), with articulations taken from three sources: (1) Trovato et al. (2017); (2) surgeon-altered; (3) the average female talus in this study.

Furthermore, a detailed examination of articulations was performed comparing the reference to the three talus shapes (Figure 7). As articular surfaces of the talus transmit motions and forces to adjacent bones, we looked at the following articular surfaces: tibial-talar, talar-navicular, and talar-calcaneal. We found that most articular surfaces deviated <1 mm, with the exception of a few cases where a small portion of points at the boundary of articulations deviated more than 1 mm. We then took the points that deviated more than 1 mm from each of the three studies, and quantified them against the reference male talus. We found that the surgeon-altered had a greater percentage of deviated points, followed by Trovato et al. (2017), and then by the average female talus in this study. In the surgeon-altered shape, we found the following percentage of deviated points more than 1 mm: tibial-talar (2%), talar-navicular (13%), and talar-calcaneal (5%). For the case of Trovato et al. (2017)'s talus, our findings revealed a comparatively lower percentage of deviated points when compared to the surgeon-altered shape; specifically, 2% in tibial-talar, 6.6% in talar-navicular, and 2.8% in calcaneal-talar. Lastly, in the average female talus in the current study, we found 2% of deviated points in tibial-talar articular surface, and negligible percentages for the other two surfaces.

## Discussion

The current study first developed an automatic groupwise registration algorithm, which was then used to register 98 tali (57 male and 41 female subjects). With this, we aimed to explore the average talus shapes and their associated principal components in both males and females. Also, we compared the average male talus with the following average talus shapes: Trovato et al. (2017), surgeon-altered and the average female talus in this study.

Earlier studies have investigated the morphology of the talus by either using anatomical landmarks to capture the characteristics of the talus (Mahato, 2011), or by performing 3D deviation analysis in a software (Islam et al., 2014). Not only would these methods involve extensive manual work, but would also be prone to errors and inconsistencies (Stagni et al., 2004, 2005; Hayes et al., 2006). In order to circumvent these issues, the current study explored the geometry of the talus using SSM with an automatic groupwise registration. As well, the current algorithm can register 98 tali within 3 days using a computer with an Intel Core processor at 3.4 GHz and a RAM of 12 GB. Thus, it is more efficient with potential for large-scale talus geometry analysis.

3D deviation analysis was performed between the first five eigenvectors, as scaled by +3σ and their means for both average male and female talus. Our findings revealed that the first two principal components can account for 50.6 and 59.1% of the male and female talus shape variations. In order to quantify their impacts, a threshold of 1 mm deviation was selected because a 1 mm talus shift can lead to 40% loss of contact with adjacent bones in talus joints (Lloyd et al., 2006). More specifically, our findings revealed that changes in PC1 can result in up to 81% of points in the male talus and 84% of points in the female talus to deviate more than 1 mm. Thus, PC1 can be considered as a scale amongst tali; its role as a major contributor has been confirmed and widely made use of in previous studies (Islam et al., 2014; Trovato et al., 2017; Zhao et al., 2019). In contrast, as the second major contributor, PC2 leads to a lower percentage of deviations greater than 1 mm: 55% of the deviations for male tali and 36% for female tali (Figure 5). The contribution of PC2 can be regarded as a local variation among most areas of articulations (e.g., curvature of tibial-talar, talar-navicular, and talar-calcaneal articular surfaces), and this effect was more obvious in male subjects (55%) than female subjects (36%) (Figure 4). In alignment with the literature, a recent study showed that the lateral talar domes can be represented using one radii, while the medial talar dome by two radii. More specifically, the medial posterior talar dome had a larger radius than that of the medial anterior talar dome (Zhao et al., 2019). Zhao et al. (2019)'s study supports the current study's findings around the deviation contour of PC2 in the area of the talar dome (Figure 5). In addition, Tümer et al. (2019) revealed that the principal variations were governed by the first three PCs and the eighth PC. When the first five PCs in the current study were compared to the shape variations in Tümer et al. (2019)'s work, most of the major shape changes could be associated with the PCs in the current study. We found that the changes in the lateral rotation of the talar head can be expressed by PC2 in the current study; the changes in the medial tubercle and the body of the talus was governed by PC3; the change in the lateral projection of the talar lateral was reflected by PC4; and the changes in the posterior talar articular surface were affected by PC2 and PC3. It should also be noted that the contributions of each PC in the current study were different from that of Tümer et al. (2019)'s work, and a size effect- aside from length- was not reported in Tümer et al. (2019)'s work. This might be attributed to the individual inter-variability and initial alignment of the dataset before registration.

**Figure 8:**
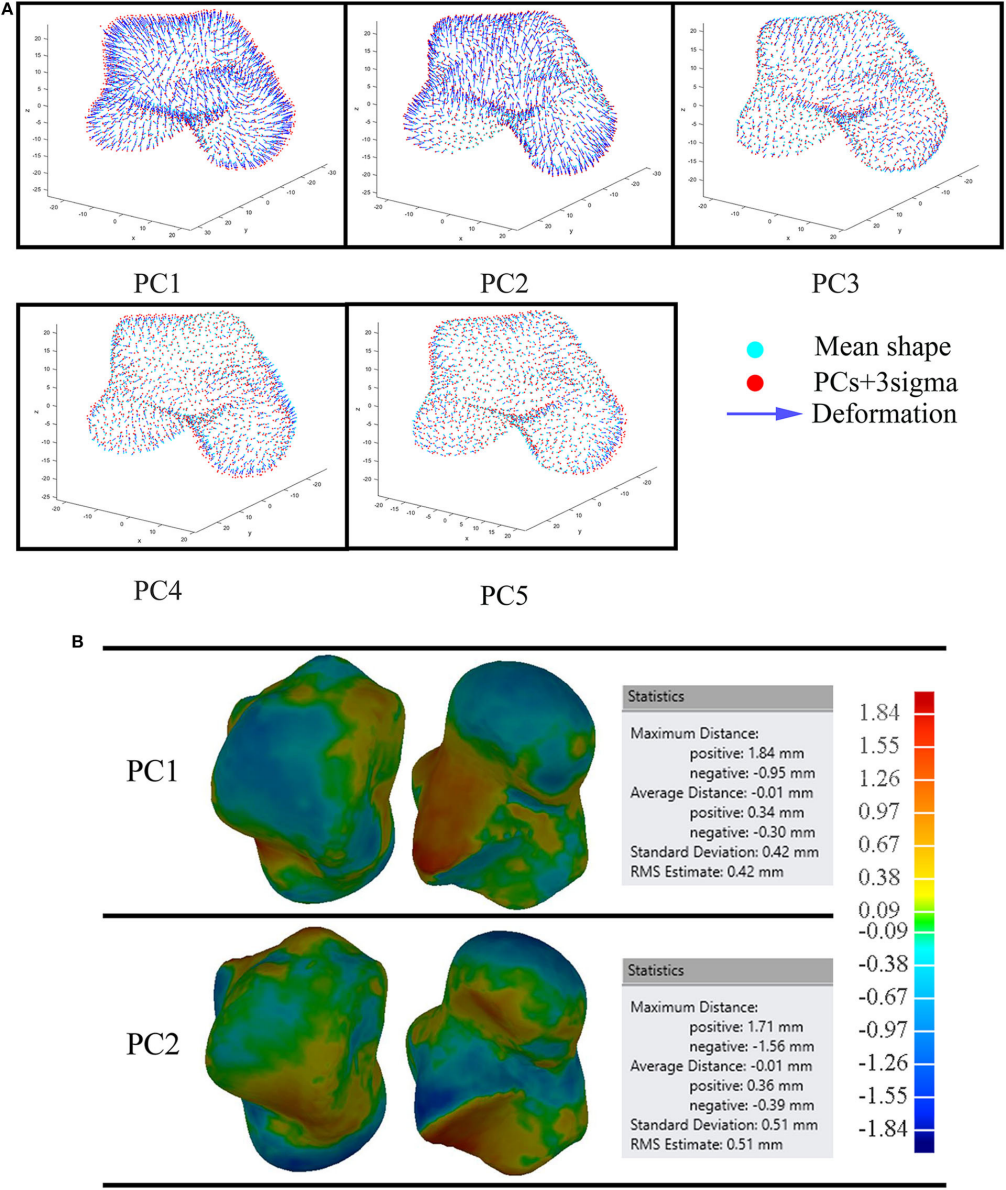
Comparison of the average male to the first five male PCA eigenvectors scaled by +3σ **(A)**; and the first two male PCA eigenvectors scaled by +3σ under the same volume **(B)**.

Earlier studies (Islam et al., 2014; Trovato et al., 2017) have operated on the assumption that the talus shape can be obtained by scaling a template talus. However, our study found that PC1 was dominated by size variation and PC2 was able to contribute to a relatively large percentage of tibial-talar and talar-navicular articular surface deformations (Figures 4, 8A). In this way, when earlier studies used the scale of only one template to represent a range of tali, they were unable to adequately account for articular surface curvature variations even though it has been concluded that the average deviation at articular surfaces between the target talus and scaled template can be <1 mm (Trovato et al., 2017). In order to exclude the contributions of scaling, the first two male PCA eigenvectors scaled by +3σ were compared to the male mean shape under the same volume (41996.3 mm^2^) (Figure 8B). We found that the first two components have a similar average distance (−0.01 mm) and standard deviation (~0.46 mm) after scaling to the same volume. More specifically, after best-fit alignment, PC1 showed relatively larger influence on the curvatures of talar-navicular and tibial-talar articular surfaces, while PC2 showed larger deviations at talar-navicular and partial talar-calcaneal articular surfaces. It is well-recognized that talar articular surfaces play a significant role in motion control, joint stability, and weight bearing. Excluding the curvature variations at articular surfaces for the talus implant might cause high pressure on articular surfaces and instability of the joint. Thus, from the perspective of geometrical accuracy, this study showed that it would be beneficial to take into consideration the first two principal components (PC1 and PC2) for a more accurate universal talus implant. However, the extent to which the articular surface curvatures of the talus implant are geometrically accurate would need further analysis of contact pressures and stresses at the articular surfaces between talus implants and the surrounding bones, compared to a biological talus.

It should also be noted that the average talus shape in the current study was smoother at the talus edges compared with surgeon-altered and Trovato et al. (2017)'s average shape. This is due to the different method the current study used to find the average talus shape, as compared to method used in the literature. In Trovato et al. (2017)'s and surgeon-altered work, the talus of a subject with the least deviation with the rest of subjects from the participant pool (*n* = 91) was selected as the average shape. However, in the current study, the average shape of the talus was calculated by averaging the coordinates of points that corresponded to each subject in the groupwise registration process.

Three paired comparisons were performed between the average male talus and the average shapes from three sources: Trovato et al. (2017), surgeon-altered and the average female talus in this study. All three comparisons showed an overall satisfactory deviation from the average male shape, at an average distance of <0.015 mm (Figure 6). This finding confirmed that the average male talus obtained in this study is similar to that of other studies, which further supports the feasibility of the groupwise registration algorithm developed in the study. With regards to the percentage of points that deviated more than 1 mm, both the Trovato et al. (2017)'s and surgeon-altered average shape showed a higher percentage of points (8%) than that of the average female shape (0.6%). When we took a closer look at deviation of points in the three articular surfaces, we found that the female talus had the least amount of deviation compared to the average male talus. It only had 2% of points deviating more than 1 mm, and the other two articular surfaces had negligible percentage of deviations. This suggests that the average female shape is geometrically similar to the average male shape, which is consistent with the findings of Trovato et al. (2017).

Similar to other studies, our study has limitations. The mean shape obtained is limited to the shapes of the 98 tali that were analyzed in the study. However, the general trend of major shape variations would be expected to keep even with a larger sample size. When the shape variations in this study are used to design a large group of talus implants, more talus samples would need to be taken into consideration to fully represent talus shape space. In addition, we only quantified the points with deviation greater than 1 mm in this paper. Were the criterion changed, more principal components would need to be considered for the implant design.

## Conclusion

In conclusion, this study presented a novel methodology using SSM to simulate the talus shape and used it to investigate the morphology of the talus based on 98 tali. By performing a 3D deviation analysis between our talus implant (which was designed from the mean shape of a SSM) and two implants from literature, the similarities we found between these implants showed the feasibility of SSM as a way of finding mean shape for talus implant design. Furthermore, we confirmed that when the average male and female talus implant shapes were scaled to the same volume, they were found to be geometrically similar. We also found that instead of using the mean shape as a scalable template directly, that the first two principal components can be taken into consideration to improve the geometric accuracy in talus implant design. However, we acknowledge that implant accuracy would also require consideration of factors in addition to geometry, such as material properties, contact characteristics of articular surfaces and joint stability. Overall, the findings in this study can provide important information on size ranges in the design of the universal talus implant, and along which directions the template should change its shape. Such knowledge would shed light on the optimal number of talus templates to design in order for most talar surgery patients to have implant templates that fit them.

## Data Availability Statement

The datasets analyzed in this article are not publicly available. Requests to access the datasets should be directed to lindsey.westover@ualberta.ca.

## Ethics Statement

Ethical approval for this human study was provided by the University of Alberta Health Ethics Review Board (Pro00026057) with a waiver of individual consent.

## Author Contributions

TL developed the algorithm and drafted the manuscript. NJ made great contributions to the improvement of the paper. SA gave substantial help with the mathematical model of the groupwise registration algorithm. ME-R contributed to the manuscript composition and interpretation of the results. LW reviewed the paper and helped the design of the work. All authors contributed significantly to this study and agreed to publish the paper in its current version.

## Conflict of Interest

The authors declare that the research was conducted in the absence of any commercial or financial relationships that could be construed as a potential conflict of interest.
